# Understanding Older People’s Readiness for Receiving Telehealth: Mixed-Method Study

**DOI:** 10.2196/jmir.8407

**Published:** 2018-04-06

**Authors:** Cornelis TM van Houwelingen, Roelof GA Ettema, Michelangelo GEF Antonietti, Helianthe SM Kort

**Affiliations:** ^1^ Technology for Healthcare Innovations Research Group Research Centre for Healthy and Sustainable Living University of Applied Sciences Utrecht Utrecht Netherlands; ^2^ Clinical Health Sciences Faculty of Medicine University Medical Center Utrecht Utrecht Netherlands; ^3^ Chronic Illnesses, Methodology of Applied Research Research Group Research Centre for Healthy and Sustainable Living University of Applied Sciences Utrecht Utrecht Netherlands; ^4^ Julius Center for Health Sciences and Primary Care University Medical Center Utrecht Utrecht Netherlands; ^5^ Building Physics and Services Department of the Built Environment Eindhoven University of Technology Eindhoven Netherlands

**Keywords:** older adults, videoconferencing, technology, path analysis, observations, community-dwelling people, UTAUT, TAM, self-efficacy, digital literacy

## Abstract

**Background:**

The Dutch Ministry of Health has formulated ambitious goals concerning the use of telehealth, leading to subsequent changes compared with the current health care situation, in which 93% of care is delivered face-to-face. Since most care is delivered to older people, the prospect of telehealth raises the question of whether this population is ready for this new way of receiving care. To study this, we created a theoretical framework consisting of 6 factors associated with older people’s intention to use technology.

**Objective:**

The objective of this study was to understand community-dwelling older people’s readiness for receiving telehealth by studying their intention to use videoconferencing and capacities for using digital technology in daily life as indicators.

**Methods:**

A mixed-method triangulation design was used. First, a cross-sectional survey study was performed to investigate older people’s intention to use videoconferencing, by testing our theoretical framework with a multilevel path analysis (phase 1). Second, for deeper understanding of older people’s actual use of digital technology, qualitative observations of older people executing technological tasks (eg, on a computer, cell phone) were conducted at their homes (phase 2).

**Results:**

In phase 1, a total of 256 people aged 65 years or older participated in the survey study (50.0% male; median age, 70 years; Q1-Q3: 67-76). Using a significance level of .05, we found seven significant associations regarding older people’s perception of videoconferencing. Older people’s (1) intention to use videoconferencing was predicted by their performance expectancy (odds ratio [OR] 1.26, 95% CI 1.13-1.39), effort expectancy (OR 1.23, 95% CI 1.07-1.39), and perceived privacy and security (OR 1.30, 95% CI 1.17-1.43); (2) their performance expectancy was predicted by their effort expectancy (OR 1.38, 95% CI 1.24-1.52); and (3) their effort expectancy was predicted by their self-efficacy (OR 1.55, 95% CI 1.42-1.68). In phase 2, a total of 6 men and 9 women aged between 65 and 87 years participated in the qualitative observation study. Of the primary themes, 5 themes were identified that could provide greater understanding of older people’s capacities and incapacities in using digital technology: (1) “self-efficacy and digital literacy,” (2) “obstacles to using technology,” (3) “prior experience and frequency of use,” (4) “sources of support and facilitating conditions,” and (5) “performance expectancy.” These 5 themes recurred in all 15 observations.

**Conclusions:**

Performance expectancy, effort expectancy, and perceived privacy and security are direct predictors of older people’s intention to use videoconferencing. Self-efficacy appeared to play a role in both older people’s intention to use, as well as their actual use of technology. The path analysis revealed that self-efficacy was significantly associated with older people’s effort expectancy. Furthermore, self-efficacy and digital literacy appeared to play a major role in older people’s capacities to make use of digital technology.

## Introduction

### Background

The increasing use of digital technology in society requires that all citizens, including older people, have digital literacy. In the Netherlands, people are gradually forced to regulate tasks online, for example, banking or government-related issues, such as tax returns or passport applications. The same applies for health care, in which digital technologies are increasingly integrated, for example, in telehealth, in which health care is delivered remotely through the use of digital technology such as videoconferencing.

On the basis of the belief that telehealth can offer a solution for the increasing number of older people with a (chronic) disease and the accompanying increasing demand for care, the Dutch Ministry of Health formulated in 2014, three ambitions with regard to the use of e-health to be achieved within 5 years: (1) 80% of chronically ill patients have direct access to (parts of) their medical record, (2) 75% of chronically ill patients and vulnerable older people who are willing and able to, actual perform self-measurements, and (3) all community-dwelling patients have the possibility to communicate via videoconferencing with their health care providers [1]. These ambitions require a major change to the current health care situation, in which 93% of the care occurs face-to-face according to a recent poll [2]. These ambitions are based on the technological possibilities of telehealth; however, they raise the question of whether patients, especially older patients, are ready for this new method of care delivery; do older people intend to use videoconferencing and what capacities do they have in using digital technology?

Olson and colleagues [3] showed that there is limited evidence that older adults are averse to using technology, but their frequency and choice of the type of technology often differ from younger adults. Older people are part of another technology generation than younger people and consequently raised with different types of technology (eg, television, radio, telephone) [4] than the technologies that are currently used in health care (eg, using the internet via PCs, notebooks, tablets, including videoconferencing and apps). To facilitate the use of new technologies, Holden and Karsh [5] emphasize the importance of end users receiving sufficient support to ensure that they feel confident in their ability to use these technologies. In health care, nurses have an important role in providing this technological support to patients to enable older people to receive telehealth [6].

Consequently, to support older people in the use of digital technology in health care, we must first understand their readiness to do so, by exploring the factors associated with older people’s intention to use digital technologies, such as videconferencing (which is a part of telehealth). Furthermore, it is relevant to explore how older people address technology in their daily life. Several studies with regard to older people’s acceptance of technology [7-10] are built on the Technology Acceptance Model (TAM) [11] and the modified version of this model, the Unified Theory of Acceptance and Use of Technology (UTAUT) [12]. The TAM [11] and UTAUT [12], however, provide neither a deep understanding of the relations and interactions between factors and nor insight into the capacities of community-dwelling older adults to use digital technology. This insight, however, is needed to understand older people’s readiness to receive telehealth.

### Purpose of the Study

This study aims to obtain a deeper understanding of community-dwelling older people’s readiness to receive telehealth by studying older people’s intention to use videoconferencing and capacities or incapacities to use digital technology in daily life as indicators. Since individual’s intention to use technology can substantially differ from their actual behavior [13], both intention and actual use of technology are examined in this study. This knowledge could benefit health care professionals’ abilities to assist older people in using technology and enable older people to benefit more from novel technology that supports them in aging in place. To achieve this, the following steps were taken and reported in this study: (1) literature review to build a theoretical framework of intention to use technology, (2) testing the framework, (3) collection of data on older people’s capacities or incapacities to use digital technology, (4) synthesis of all results, and (5) conclusions and implications for older people’s readiness to use telehealth.

**Figure 1:**
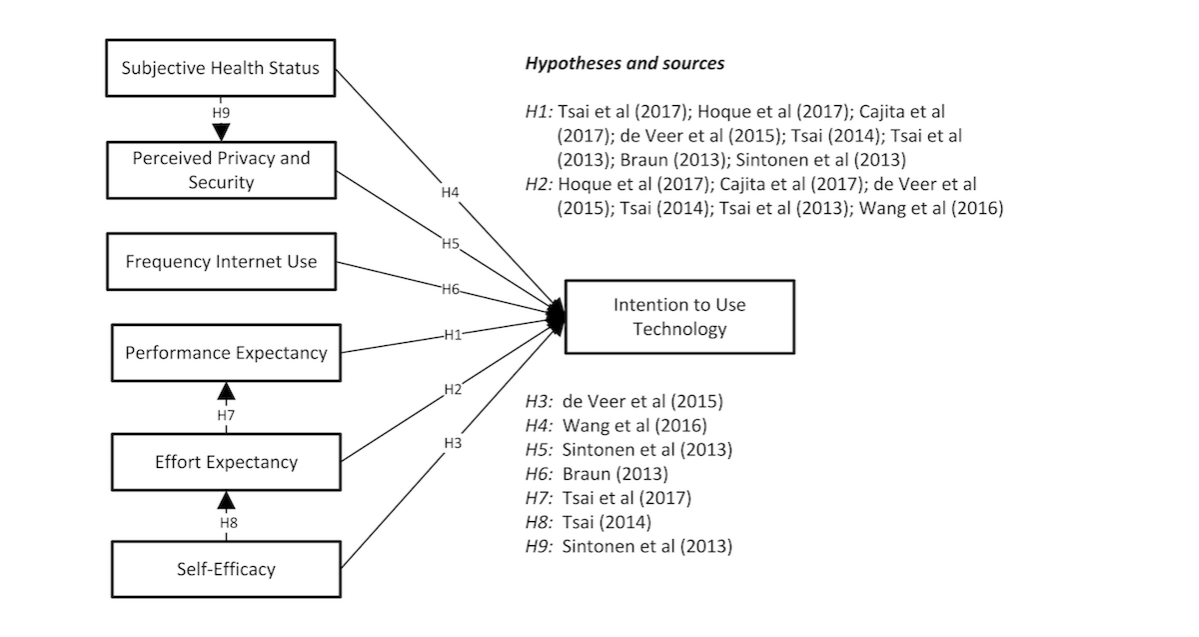
Theoretical framework. This framework displays the factors associated with older people’s intention to use technology. Each hypothesis is based on prior research, as shown. H=hypothesis.

### Literature Review—Older People’s Intention to Use Technology and Associated Factors

In 2017, a literature search was performed to build a theoretical framework of older people’s intention to use technology and associated factors. Therefore, the search terms “older people,” “technology,” “intention,” and “factors,” as well as alternative terms, such as “seniors” and “associations,” were used for a search in CINAHL, Google Scholar, PsycINFO, MEDLINE/PubMed, ScienceDirect, Scopus, and Web of Science. To scope the literature review, inclusion criteria were as follows: (1) target group with a median or mean age of 65 or older, (2) publication date less than 10 years ago, (3) peer-reviewed original research, (4) quantitative studies in which “intention to use technology” was tested as a dependent variable, and (5) studies written in English. The process and results of the literature search, in terms of search strings, number of hits, and number of selected studies, are shown in the Multimedia Appendix 1.

### Theoretical Framework of Intention to Use Technology

Of the 249 studies that were found in September 2017, only 29 studies met the criteria. After duplicate studies were filtered out, 11 studies remained [8,14-23], and these 11 studies were used to build the theoretical framework on older people’s intention to use technology (see Figure 1). On the basis of the 11 included studies, the theoretical framework shows six predictors of older people’s intention to use technology: performance expectancy, effort expectancy, self-efficacy, subjective health status, perceived privacy and security, and frequency of internet use. The operationalization of these predictors is presented in Table 2 in the Results section. All hypotheses with regard to older people’s intention to use technology and the related sources of evidence are illustrated in Figure 1.

## Methods

### Design

A mixed-method triangulation design [24] was used, including a cross-sectional survey study (phase 1), generating quantitative data concerning older people’s intention to use videoconferencing, and an observational study (phase 2), yielding qualitative data concerning their actual use, by observing their capacities in using technology in daily life. In phase 1, we focused on “videonferencing,” a relatively new technology (in health care), that is often used in telehealth services. To observe a representative sample in phase 2, we focused in this phase on more traditional, commonly used technologies. If we adhered to videoconferencing, we could have observed only those older people who use videoconferencing, which might have introduced a selection bias. Both insights, intention to use and actual use of technology, are important to understand older people’s readiness to receive telehealth.

### Phase 1: Cross-Sectional Study on Older People’s Intention to Use Videoconferencing

#### Setting and Participants

Participants were recruited in September 2012 for the cross-sectional study. Older people were invited to fill out a paper version of the survey through 2 patient advocacy organizations, 2 senior social clubs, 5 health care organizations, and a senior information day in Utrecht. Additionally, to reach a large group of potential participants, a Web-based panel of approximately 2000 clients was invited to fill out an online version of the survey.

Two inclusion criteria, (1) independently living at home and (2) being 65 years of age or older, were maintained. To estimate the required sample size, we followed the rule of thumb for multiple regression analysis, with the purpose of building a prediction framework: maintain a ratio of 10 positive cases in the dataset to 1 predictor variable in the full path analysis [25]. Since the full path analysis contains 6 predictors, a sample with at least 60 positive cases was required. The dependent variable “intention to use” (measured on a 5-point scale, with 1=totally disagree, 2=disagree, 3=neutral, 4=agree, and 5=totally agree) was used to calculate the number of positive cases by labeling participants with a response from 1 to 3 as “non-cases” and participants with a response from 4 to 5 as “positive cases,” resulting in 70 positive cases.

#### Cross-Sectional Survey

We collected data using a survey to test the hypotheses illustrated in the theoretical framework. The outcome measured was set as “intention to use videoconferencing,” aligned with the ambitions of the Dutch ministry in which the use of videoconferencing is an important part of telehealth.

The survey included items covering the following topics: demographic questions (eg, age, gender, and educational level); health-related questions (eg, health status) based on Czaja et al [26]; technology experience in daily life (with, eg, internet, computer) based on Czaja et al [27]; and older people’s perception of videoconferencing (eg, performance expectancy, effort expectancy, and intention to use videoconferencing) based on Chang and Hsu [28] and Gagnon et al [29]. All constructs regarding older people’s perception of their health and perception of videoconferencing were measured with multiple statements.

Table 2 lists all constructs (predictors and dependent variables) and related statements that were used to test our theoretical framework. The survey items that were based on previously developed and used questionnaires were translated from English into Dutch and cross-translated. Subsequently, the content of the survey was discussed with experts in aging and technology who were selected from our network and pilot-tested among a representative group of older people to determine the readability and comprehensibility.

#### Statistical Analysis

Missing values were substituted using the 5-time multiple imputation method to reduce bias [30]. The results of the statistical analysis of each of the 5 imputed dataset were pooled using Rubin’s rule [31].

The internal consistency of the constructs (eg, self-efficacy, performance expectancy) was assessed with Cronbach alpha, considering Cronbach alpha values between .70 and .95 to be “good” [32]. For our multilevel regression analysis, the variable “frequency of internet use” was dichotomized, using a data-driven method to select an appropriate cut-off point. In the survey, participants were asked: “on average, how many hours per week do you use the internet?,” whereby, 0=not, 1=0 to 1 hours, 2=1 to 5 hours, 3=5 to 10 hours, 4≥10 hours. The cut-off point was set at 2, meaning 0=less than 5 hours a week and 1=5 or more hours per week.

All hypotheses, as illustrated in our theoretical framework (Figure 1), were tested using a path analysis approach [33]. In the full path analysis, 4 outcome variables, that were interconnected, were tested at once (see Figure 1): Outcome 1 “intention to use videoconferencing” predicted by (1) “performance expectancy,” (2) “effort expectancy,” (3) “self-efficacy,” (4) “subjective health status,” (5) “perceived privacy and security,” and (6) “frequency of internet use”; Outcome 2 “performance expectancy” predicted by “effort expectancy”; Outcome 3 “effort expectancy” predicted by “self-efficacy”; and Outcome 4 “perceived privacy and security” predicted by “perceived privacy and security.” In this way, the effects of group-level predictors were deliberately confounded with the effects of the group variables, thus upholding the hierarchical structure. As such, the effects of both levels of variables could be estimated. All three outcome variables and accompanying predictors in the path analysis were based on the literature mentioned in the introduction. A significance level of .05 was used to determine whether predictors had a significant association with the dependent variable.

**Figure 2:**
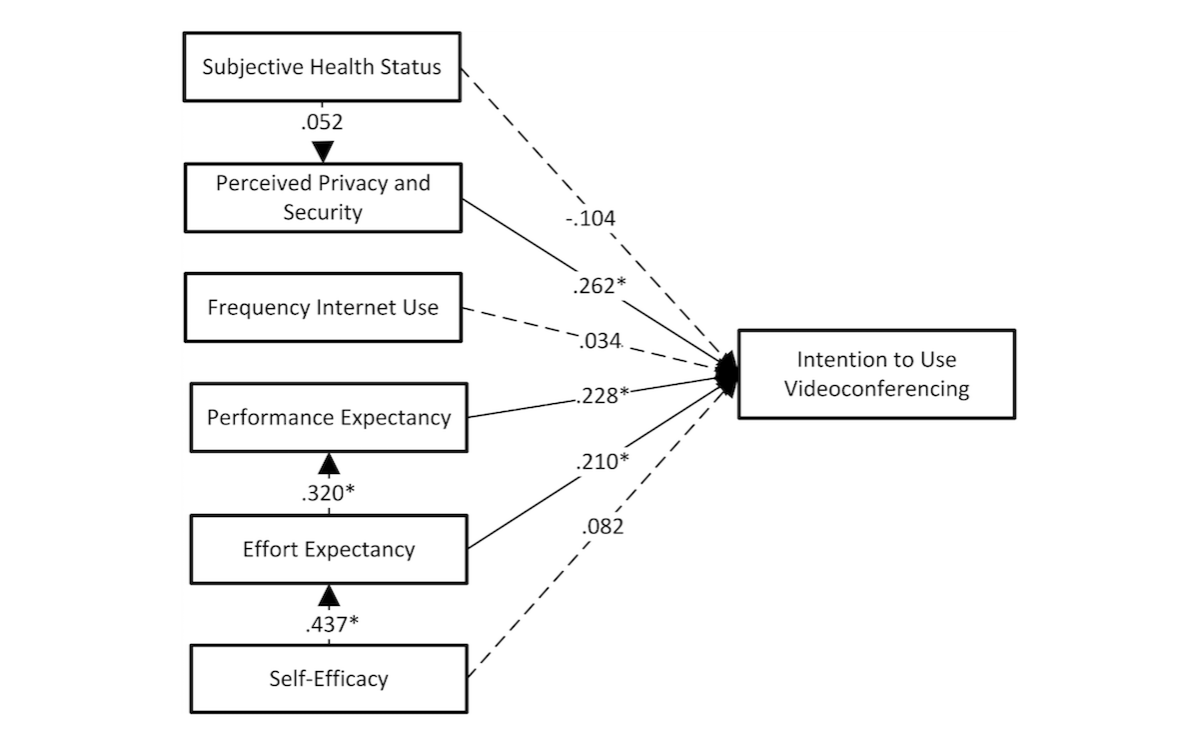
Older people’s (n=256) intention to use videoconferencing and associated factors. Unstandardized regression coefficients are shown, derived from the path analysis. Estimates were pooled from the results of the analysis of 5 imputed datasets using Rubin’s rules. *Significant association, using a significance level of .05 (dotted-line indicates nonsignificance).

**Figure 3:**
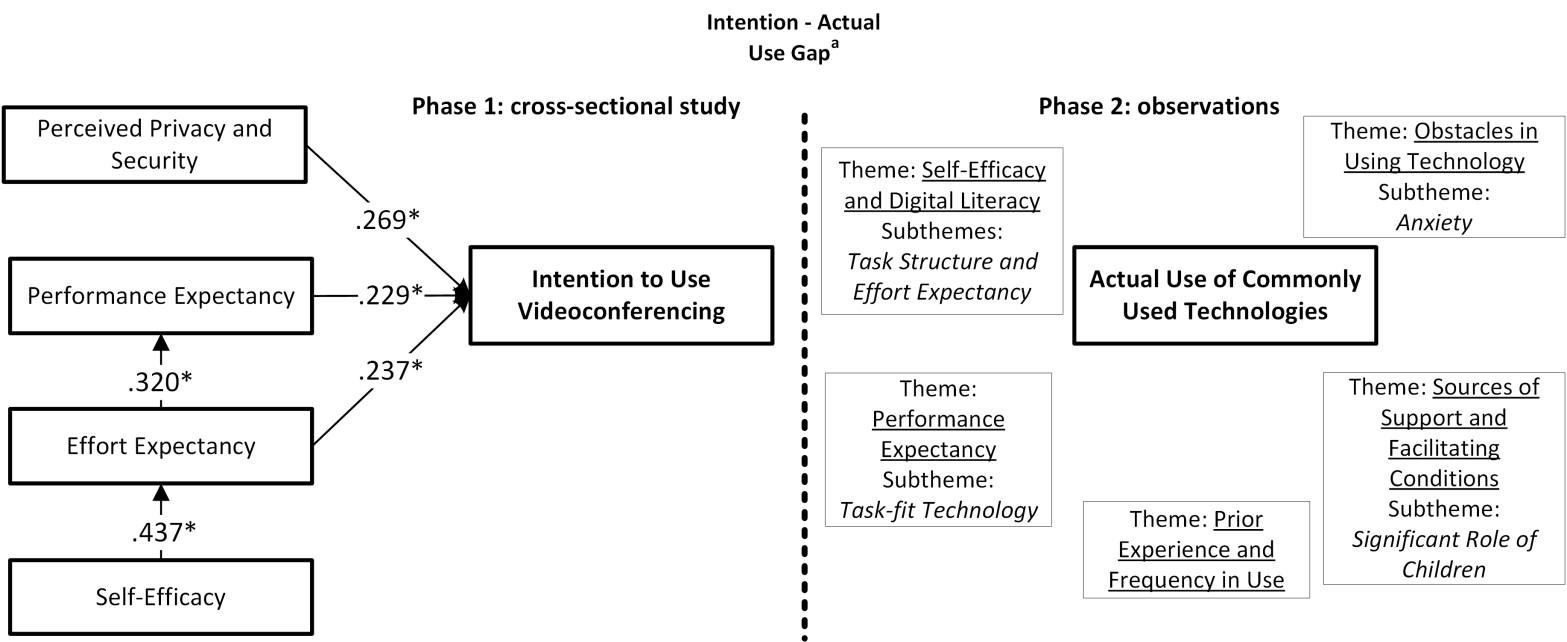
Understanding older people’s intention to and actual use of technology. A mixed-method framework of a multilevel regression path analysis (n=256) and qualitative observations (n=15). *Significant (alpha .05) associations; unstandardized regression coefficients are shown. The letter “a” denotes that this “Internet—actual use gap” was based on prior research.

Additionally, starting with the full path analysis (Figure 2), a backward selection procedure was performed; at each step, the variable with the highest *P* value was excluded first. Simultaneously, at each step, the goodness of fit of the framework was examined using the Akaike Information Criterion (AIC) [34] to assess the performance of each model compared with the initial framework. Following this procedure, a final path analysis (Figure 3) was reached with only significant (significance level of .05) predictors and using the AIC as a threshold. In this final framework, the themes derived from the qualitative data were included.

We used the statistical package R (version 3.4.2; 2017-09-28; The R Foundation for Statistical Computing) for the path analysis. All other statistical analyses were performed using SPSS (IBM Corp Released 2016, IBM SPSS Statistics for Windows, Version 24.0. Armonk, NY: IBM Corp).

### Phase 2: Observations on Using Technology in Daily Life

#### Qualitative Approach and Research Paradigm

Given the relative lack of exploratory studies on community-dwelling older people’s capacities or incapacities to use technology, we performed a conventional content analysis [35] on the qualitative data obtained from observations of older people using digital technology at their home. Our approach was constructivist, using an interpretative phenomenological epistemology [36] based on the notion that there is not one “truth” in regard to the phenomenon of technology use.

The observations were executed by the third author and by third-year bachelor’s degree-level students with backgrounds in nursing, health care management and Cesar exercise therapy. Before their observations, these students received training from our research team on how to perform observations. None of the observers were known to the participants before the observations being performed. All observations were discussed by our multidisciplinary research team. As members of the research team, the first author of this paper (CvH) has a background in nursing, sociology, and nursing education; the second author (RE), in nursing, nursing science, and epidemiology; the third author (MA), in human movement science and nursing education; and the fourth author, in medical biology, built environment, and gerontechnology (HK).

#### Context, Setting, and Sampling

In 2012, between September and December 2012, we conducted the study among community-dwelling older people (65 years of age or older). The sampling started in phase 1, along with the survey, and participants were informed about the opportunity to also volunteer in phase 2 (the observations). When the participants were willing to participate in phase 2, they filled out a form with contact details, which they returned to the research team together with their completed survey. For phase 2, the same inclusion criteria were held as in phase 1: (1) independently living at home and (2) being 65 years of age or older. However, we also selected based on both “experience with a computer” and “experience without a computer,” with the aim of being able to observe participants with and without computer experience. After 15 cases were observed, theoretical saturation was reached; sufficient data were collected to understand the concepts of our study.

#### Data Collection and Processing

To facilitate consistency over different sets of observations, a list of day-to-day technological tasks was composed before the data collection. The tasks were developed by the third and last authors during a 2-day workshop with our American research partners (details described under “Acknowledgments”), which resulted in the following 8 categories: (1) computer basics (eg, “create a new folder on your desktop”), (2) email (eg, “send an email with an attachment”), (3) use of the internet (eg, “show a map of your town”), (4) television (eg, “change the volume of your TV”), (5) mobile phone (eg, “show how to save a contact in your contact list”), (6) household (eg, “make popcorn in a microwave”), (7) health (eg, “show how to use a digital scale”), and (8) videoconferencing (eg, “show how to start videoconferencing with your nurse”).

Without a specific time constraint, the observant followed this list of tasks and encouraged the older adult to accomplish the task independently. The older people could ask for a hint when they could not proceed with the task. The observant encouraged the older people to think aloud during the performance of their technological tasks. During the direct observations, notes were made using a form with space for notes for each of the tasks and blank space for other possible remarkable occurrences. These notes were used during the iterative analysis process.

All observations were audio-recorded and lasted 1 hour on average. The audio-recordings were transcribed and anonymized. All transcripts were stored, coded, and analyzed in MAXQDA (software for qualitative data analysis, 1995-2016, VERBI Software—Consult—Sozialforschung GmbH, Version 12.2.1, Berlin, Germany).

#### Data Analysis

Data analysis followed the steps for conventional content analysis as outlined by Hsieh and Shannon [35], a method to describe a phenomenon—in this case, the daily use of digital technology by older people. Through an iterative process of coding, by discussing findings in the light of the literature, the research team identified and described the most prevalent themes with regard to older people’s day-to-day use of technology. The concepts derived from our theoretical framework in (Figure 1) were used as “sensitizing concepts,” defined as “interpretive devices and as a starting point for qualitative research” [37]. In addition to the concepts derived from Figure 1, the contextual factors in the use of technology, as described by McFarland and Hamilton [38], were also used as a starting point for analysis (eg, task structure, prior experience, other’s use).

Although these 2 frameworks were used, we conducted an open, inductive analysis, starting with open coding. To enhance trustworthiness, CvH and MA coded the verbatim transcripts independently, with a focus on the sensitizing concepts and the main question: “How do older people struggle with digital technology use and what supports them?” The first round of coding by two of the authors (CvH and MA) resulted in 1022 coded text segments. Then, these open codes were discussed among all authors to organize and group the codes into meaningful clusters. After this discussion, 157 text segments were considered irrelevant. The remaining 881 text segments and related codes were clustered and categorized. We searched for themes that occurred in each observation with all participants. Eventually, we achieved consensus on the primary themes observed in the data.

In the last phase, definitions for each theme were developed and provided with illustrative examples or quotations from the data. Quotations in this study were translated from Dutch into English. During the whole analysis, we kept in mind that we were looking for information that could eventually benefit nurses’ in assisting older people to use technology in health care. To illustrate the qualitative results, the themes and subthemes were drawn in Figure 3.

### Ethical Approval

This research was conducted following Dutch human subject regulations. Since the Dutch Medical Research Involving Human Subjects Act did not apply to either phase 1 or phase 2 of this study, no official ethical approval was required. Nevertheless, all necessary precautions were taken to protect the anonymity and confidentiality of our participants. The Dutch Medical Research Involving Human Subjects Act applies to medical research “if there is an infringement of the physical and/or psychological integrity of the subject” [39].

Cliëntenbelang Utrecht (an organization that defends the interest of health care clients) approved the study and provided access to the client panel. All participants were informed with a letter containing information about the purpose of the study. Participants were informed that their participation was voluntary, that they were free to decline or discontinue their participation at any time and that their responses were processed anonymously and only used for research purposes. No person identifying information was collected.

## Results

### Phase 1: Cross-Sectional Study on Older People’s Intention to Use Videoconferencing

#### Characteristics of Study Population

In total, 288 older people filled out the questionnaire on paper or online. Of these individuals, 22 were excluded since they were younger than 65 years of age. Of the 256 cases left, 50.0% (128/256) were male and 50.0% (128/256) were female, with a median of 71 years (Q1-Q3 67-76). A minority (13.7%, 35/256) of participants had experience with videoconferencing, of whom approximately half had less than 1 year of experience, while the other half had more than 1 year. The majority (71.1%, 182/256) completed an average or high level of education. Of the 256 cases, 21.1% (54/256), missed one or more questions that were used for this study. Their missing values were substituted using the 5-time multiple imputation method. All demographic details of the participating older people are listed in Table 1.

#### Descriptive Results and Consistency of the Research Constructs

The internal consistency of the 6 constructs was “good,” with a Cronbach alpha of more than .70 [32]. All grouped items and accompanying median scores, 1st and 3rd quartile ranges, and Cronbach alphas are presented in Table 2.

#### Results of the Path Analysis

Using a significance level of .05, the multilevel path analysis revealed that 5 of the 9 hypotheses regarding older people’s perception of videoconferencing were supported. On level 1, older people’s intention to use videoconferencing was significantly predicted by their performance expectancy (odds ratio [OR] 1.26, 95% CI 1.13-1.39), effort expectancy (OR 1.23, 95% CI 1.07-1.39), and perceived privacy and security (OR 1.30, 95% CI 1.17-1.43). In our sample, self-efficacy (OR 1.09, 95% CI 0.94-1.23), subjective health status (OR 0.90, 95% CI 0.79-1.01), and frequency of internet use (OR 1.03, 95% CI 1.42-1.68) were not significantly associated with older people’s intention to use videoconferencing.

On level 2, older people’s performance expectancy was predicted by their effort expectancy (OR 1.38, 95% CI 1.24-1.52). On level 3, their effort expectancy was predicted by their self-efficacy (OR 1.55, 95% CI 1.42-1.68). Our last hypothesis, on level 4, was not supported: older people’s perceived privacy and security was nonsignificantly predicted by their subjective health status (OR 1.05, 95% CI 0.95-1.16). The complete path analysis and unstandardized regression coefficients, from which the ORs were derived, are illustrated in Figure 2, with intention to use videoconferencing as the main outcome variable.

**Table 1:** Demographic characteristics of participating older people (n=256; paper participants [n=70] and online participants [n=186]). N/A: not applicable.

Characteristics		n (%)
**Gender**		
	Male	128 (50.0)
	Female	128 (50.0)
**Age by category (in years)**		
	65-74	182 (71.1)
	75-84	67 (26.2)
	>85	7 (2.7)
Median age=71 (Q1-Q3=67-76)		N/A
**Experience with the use of video conferencing**		
	Yes	35 (13.7)
	No	221 (86.3)
**Highest completed educational level**		
	Lowest (primary education)	10 (3.9)
	Low (lower secondary education)	57 (22.3)
	Average (general or vocational upper secondary education)	70 (27.3)
	High (postsecondary nontertiary education)	119 (46.5)

**Table 2:** Constructs of the path analysis: internal consistency and median scores. N/A: not applicable.

Construct and related items		Cronbach alpha^a^	Median (1st quartile-3rd quartile)
**Subjective health status (predictor variable)**		.87	3.0 (2.3-3.3)
	1. In general, I would say my health is^b^		3.0 (2.0-3.0)
	2. Compared with other people of my age, I would say my health is^c^		3.0 (2.0-3.0)
	3. How satisfied are you with your present health?^c^		4.0 (3.0-4.0)
**Performance expectancy (predictor and outcome variable)**		.72	3.3 (3.0-4.0)
	1. By using videoconferencing, I can live longer in my own home independently^d^		4.0 (3.0-4.0)
	2. The use of videoconferencing will give me more freedom^d^		3.0 (3.0-4.0)
	3. The use of videoconferencing will enhance my self-reliance^d^		3.0 (3.0-4.0)
**Effort expectancy (predictor and outcome variable)**		.85	3.8 (3.0-4.0)
	1. I think videoconferencing will be clear and easy to use^d^		4.0 (3.0-4.0)
	2. Videoconferencing will be easy to operate and use^d^		4.0 (3.0-4.0)
	3. Videoconferencing will be easy to learn^d^		4.0 (3.0-4.0)
	4. Videoconferencing will have a clear guide for operation^d^		4.0 (3.0-4.0)
**Self-efficacy (predictor variable)**		.77	4.0 (3.4-4.2)
	1. I am confident enough to use videoconferencing^d^		4.0 (3.0-4.0)
	2. Given an appropriate training, I will have the ability to use videoconferencing^d^		4.0 (3.0-4.0)
	3. I possess the necessary skills to learn how to use videoconferencing^d^		4.0 (3.0-4.0)
	4. I am afraid I will not learn how to use videoconferencing^e^		4.0 (4.0-5.0)
	5. I think I will find it hard to acquire the necessary skills to use videconferencing^e^		4.0 (3.0-5.0)
**Perceived privacy and security (predictor and outcome variable)**		.79	3.3 (2.8-3.7)
	1. My feeling of security is higher with the use of videoconferencing^d^		3.0 (3.0-4.0)
	2. With the use of videoconferencing my feeling of security will be higher^d^		3.0 (3.0-4.0)
	3. The possibility of immediate contact with a health care professional will give me a safe feeling^d^		4.0 (3.0-4.0)
	4. The use of videoconferencing is confidential^d^		3.6 (3.0-4.0)
	5. I will have no problems with the idea that videoconferencing consultations are saved^d^		3.0 (2.0-4.0)
	6. The use of videoconferencing will not influence my feeling of privacy^d^		3.0 (2.4-4.0)
Frequency of internet use^f^ (predictor variable)		N/A	N/A
**Intention to use videoconferencing (outcome variable)**		.76	3.5 (2.8-4.0)
	1. I am willing to use videoconferencing to complement my traditional care^d^		3.0 (2.4-4.0)
	2. I have the intention to use videoconferencing routinely to receive care^d^		3.0 (2.0-4.0)
	3. I intend to use videoconferencing when this is necessary to receive care^d^		4.0 (3.0-4.0)
	4. After an appropriate training, I am willing to use videoconferencing^d^		4.0 (3.0-4.0)

^a^Cronbach alpha between .70 and .95 is “good” [32].

^b^Likert scale ranging from 1=“poor” to 5=“excellent.”

^c^Likert scale ranging from 1=“not satisfied at all” to 5=“very satisfied.”

^d^Likert scale ranging from 1=“totally disagree” to 5=“totally agree.”

^e^Likert scale ranging from 1=“totally agree” to 5=“totally disagree.”

^f^Participants were asked: “on average, how many hours per week do you the internet?” 0: not, 1: 0-1 hours, 2: 1-5, 3: 5-10 hours, 4: >10 hours. For the path analysis, this variable was dichotomized, using a data driven method to select an appropriate cut-off point. The cut-off point was set at 2, meaning 0=less than 5 hours a week and 1=5 or more hours per week.

### Phase 2: Observations on Using Technology in Daily Life

#### Characteristics of Observed Older People

Of the survey population of 256 older people, 16 older people volunteered to take part in phase 2 of the study: with observations conducted at their homes while they executed technological tasks. The quality of one of the audio recordings was too poor to be able to create a verbatim transcription, leaving 15 observations suitable for qualitative analysis.

Among the observed older people were 6 men and 9 women. Their age ranged from 65 to 87 years (mean=73.21, SD=6.59, 1 missing value). Of the participants, 7 had low levels of education (lower secondary education), 2 received average-level education (general or vocational upper-secondary education), and 4 completed high-level education (bachelor’s degree or higher, 2 values missing). Approximately half of the participants did not have a computer (n=7), while the other half did use a computer (n=8).

#### Understanding Older People’s Capacities to Use Technology

In all, 5 primary themes were identified that could help us understand older people’s capacities and incapacities in using digital technology (ordered by frequency of occurrence): (1) “self-efficacy and digital literacy,” (2) “obstacles to using technology,” (3) “prior experience and frequency of use,” (4) “sources of support and facilitating conditions,” and (5) “performance expectancy.” These 5 themes were observed among all 15 participating older people and included 865 of the 1022 coded text segments. Within these 5 primary themes, several subthemes were identified, which are described below and illustrated with exemplary quotations.

##### Theme 1: Self-Efficacy and Digital Literacy

In phase 2, “self-efficacy and digital literacy” was the most prevalent theme that appeared to play a role in the day-to-day use of technology by the older people we observed. “Self-efficacy” refers to an individual’s belief in his or her ability to accomplish a certain task in a specific situation [40]. We observed many situations in which older people expressed low self-efficacy regarding technology use, but approximately the same number of situations occurred in which high self-efficacy was expressed. The following conversations between a man and his wife illustrate the low self-efficacy of the man and obstacles he experienced with his computer. The conversation between the man and his wife started after the participant (man) was asked to open his email, and to be able to use his email, the participants had to turn on his computer first:

Man: [With e-mails] I do nothing. I’m a “digital illiterate.” [To his wife:] You always have a note attached, don’t you? It’s not there, so I know nothing.

Wife: Can you turn it on, or not?

Man: I don’t know, something with green, whether it’s the right or left button. Was it something green?

Wife: Just try it.

Man: Nothing happens.

Wife: You have to push it longer. Do you hold it the other way around?

Man: Nothing happens at all.

Wife: You do have to hold it longer, the red button.

Man: There is nothing red at all.

Wife: No, it isn’t red yet, you have to hold it longer.

Participant 2, male, 81 years

The theme of self-efficacy occurred in a variety of ways during the observations; older people believed that they were not able to accomplish certain technological tasks (low self-efficacy), but discovered that they actually were able to do so or could do so after a small suggestion on how to proceed. Further, we observed older people who could explain very clearly how an application or device worked and were proud that they possessed the right skills, for example:

I think it’s already good that I am able to open my e-mail and send e-mails back.

Participant 9, female, 72 years

Another recurrent observation was older people who kept very strictly to the things that they had learned and stayed away from abilities outside of their knowledge. For example, one participant said the following about his email application:

I never look over there, I just do everything I have learned.

Participant 2, male, 81 years

The same participant added:

Outside of that [email application], I become nervous.

Participant 2, male, 81 years

“Digital literacy” refers to “a large variety of complex cognitive, motor, sociological, and emotional skills, which users need in order to function effectively in digital environments” [41]. During the observations, while older people were executing technological tasks, almost all participants experienced their limited digital literacy. This limited “digital literacy” impacted their technology use in several ways: (1) the functionality of a device was only partially used since participants did not understand how to use several functions or how to use the required buttons and (2) when a new device was bought, everything had to be learned from the start, as exemplified by one of the participants:

But I notice that I'm not so good at electrical devices, so this [task] has to go very slow. [...] Yes, I remember, I was in the store and I touched it but I did not know how it worked anyway. I actually felt like a “dummy.” And I was reluctant, but he [salesman] explained me how to put that thing on/off. He said, “try to do it.” And there are also things that I could execute at that moment, but not anymore [once at home]. And then I have to ask again how it works.

Participant 9, female, 72 years

###### Task Structure

Within the theme of self-efficacy and digital literacy, two subthemes were identified: “task structure” and “effort expectancy.” Regarding the task structure, which is referred to in the literature as the extent to which a task is nonroutine and varied [38], we observed several older people who used the functionality of a device only partially and, as a result, did not benefit from all the possibilities the device offered. One male participant, for example, stated that he only *reads* emails but never responds:

In the past, I’ve had to type sometimes, but that’s way too difficult, so I only read e-mails. As long as I have her [his wife], she does that.

Participant 2, male, 66 years

Another participant explained that she only uses her cell phone in specific situations:

I only use it [cell phone] when I visit my son. When I sit in the train, I call my son and ask him to pick me up. But besides that, I never take my phone outside.

Participant 13, female, 70 years

###### Effort Expectancy

We also gained insight into the role of “effort expectancy,” defined in the literature as “the degree of ease associated with consumers’ use of technology” [12]. Several participants were complaining about the nonease of use of the technologies they used while executing the technological tasks. One woman, for example, talked about the difficulty of saving a number in the contact list of her cell phone:

...[in order to save a contact] I have to search a lot, but I will get it done. Please wait, this is very illogical [...]. Very illogical. I hope future devices are smarter.

Participant 11, female, 76 years

Another example of how effort expectancy plays a role in the use of technology came from a participant who prepared himself for executing a task with his cell phone. Seemingly easy functions can already be difficult:

I first have to turn it [cell phone] on. That’s always a bit tricky. Especially my wife has difficulties finding that on button.

Participant 7, male, 74 years

##### Theme 2: Hurdles to Using Technology

###### Obstacles

Older people experience all kinds of obstacles to using technology, also referred to in the literature as “barriers” [42,43], which are elements that hamper their use of technology. We observed obstacles in diverse categories. At first, technical obstacles presented themselves, for example, the disruption of internet service, a broken button, a slow-running computer, or a stuttering connection while videoconferencing.

Furthermore, we observed obstacles in the category “limited digital literacy,” for example, unable to find the cursor (of the mouse), getting confused after updates, or not knowing how to use the internet. The third category included more personal use-related barriers, for example, prefers to read the news in the newspaper instead of on an iPad, forgets his or her password very often, or having resistance toward social media, as expressed by one participant:

Wearing a personal alarm around my neck is fine with me, but [...] Facebook and whatever else there is, is another reality beyond my sensory reality.

Participant 11, female, 76 years

###### Anxiety

Additionally, in 12 cases, the subtheme “anxiety” was identified as an obstacle to using technology. McFarland and Hamilton [38] use a slightly different term, namely “computer anxiety,” which they describe as “an individual’s uneasiness or apprehension toward computers.” During our observations, a variety of anxiety-inducing sources arose related to the use of technology, including (1) receiving spam, (2) experiencing system updates, (3) losing written text, (4) damaging a device, (5) fearing the use of technology in general, (6) fearing microwave radiation, (7) fearing inadequate privacy protection, (7) feeling unsafe using the internet, and (8) fearing online scams or cyber criminals. Regarding the last 2 obstacles mentioned, one participant expressed her fear of online banking:

One hears so much...things that can go wrong with online banking. I dedicated myself to, if possible, only do online banking when one of my two children is with me.

Participant 9, female, 72 years

All anxiety sources mentioned above hampered the participants’ use of technology.

##### Theme 3: Prior Experience and Frequency of Use

While executing technological tasks, the theme of prior experience and frequency of use was exhibited by all participants. We observed people with much experience and little experience, as well as participants who told to have a device but reported never using it (eg, did not use their cell phone since they already had their landline telephone).

The capacities and incapacities regarding technology use seemed to be associated with older people’s experience in the past and/or their frequency in use. Some participants said they were glad that they learned to work with computers during their working career. Others did not and had to learn everything from the start. Their limited experience hampered their capacity to accomplish technological tasks:

I really don’t know how it works, I just have it [computer].

Participant 14, female, 68 years

In several cases, participants had forgotten how to accomplish a specific task since they reported only doing it once or twice in the past.

In contrast, more prior experience was clearly supportive:

This isn’t really complicated, since I already have been working with that computer for 2 years now.

Participant 6, male, 86 years

According to the participants in this study, capacity in using technology is a matter of experience and practice.

##### Theme 4: Sources of Support and Facilitating Conditions

###### Facilitating Conditions

When participants had to overcome obstacles to technology use, they reached out to various sources of support. These sources of support are part of the “facilitating conditions,” defined as “consumers’ perceptions of the resources and support available to perform a behavior” [12]. A variety of sources of support came up, such as following a computer course via SeniorWeb, a very important Dutch forum according to one of the participants:

SeniorWeb is really important, but I wonder if people take that step. [...] For me, it’s amazing to see that, myself included, my family, brothers and sisters encounter the same [obstacles].

Participant 8, male, 65 years

Further sources of support that were mentioned by our participants were manuals, helpdesks, installers, and persons, often including partners, friends, children, and grandchildren.

###### Significant Role of Children

Children and grandchildren play a significant and diverse role in the use of technology by older people; they appeared to function as a motive or incentive to start using technology, for example, since technology offers the ability to communicate more easily (and over distance). Subsequently, children help their parents in purchasing, installing, and using technology. The active support of children solves issues in the use of technology on the one hand while on the other hand, it might cause older people to maintain their lack of technology skills. When they struggle with technology, some older people wait for their children to solve it:

I’m not good at saving a number. My grandchildren always come to do that.

Participant 14, female, 68 years

Another example came from a woman who was asked to send an email to multiple persons:

My children once said, “just put all those names here” but I don’t have a clue of the meaning of all this. [

Participant 13, female, 70 years

##### Theme 5: Performance Expectancy

###### Performance Expectancy

“Performance expectancy,” a well-known construct in technology-acceptance theories, refers to “the degree to which using a technology will provide benefits to consumers in performing certain activities” [12]. Our participants mentioned benefits in various categories: (1) leisure, for example, playing games, reading books, and using street view; (2) increasing communication possibilities, for example, (also mentioned earlier) with family or nurses; and (3) aging in place, as illustrated by the following statement:

I’m already thinking of what do I need to have? What do I have to do, so in about 10 years...what do I need in order to be able to live at home as long as possible?

Participant 8, male, 65 years

###### Task-Technology Fit

Within the performance expectancy theme, a recurrent statement was that the technology must fulfill a need. This idea is close to the construct of “task-technology fit,” which refers to the assumption that “performance impacts will result from task-technology fit—that is, when a technology provides features and support that ‘fit’ the requirements of a task” [44]. Sometimes an event occurred in the lives of our participants that caused a certain technology to suddenly fit their needs, as illustrated in the following statement:

This tablet...I bought it because I like to read. And now, my eyes have become so bad that I can’t read books anymore [from paper].

Participant 9, female, 72 years

The same participant explained her motivation to purchase a computer:

I had to do financial matters, and at that moment, I took a computer.

## Discussion

### Principal Findings

In this study, 7 significant associations regarding older people’s perception of videoconferencing were found. Older people’s (1) intention to use videoconferencing was predicted by their performance expectancy, effort expectancy, and perceived privacy or security; (2) their performance expectancy was predicted by their effort expectancy; and (3) their effort expectancy was predicted by their self-efficacy. In other words, whether older people intend to use videoconferencing depends on their expectations of the usefulness of this application, their expectations of how easy it is to use videoconferencing, and their confidence whether their privacy and security is protected when using videoconferencing.

Self-efficacy did not appear to be a significant predictor of older people’s intention to use videoconferencing. However, the multilevel regression analysis made it possible to identify multiple associations within the path analysis and showed us that self-efficacy significantly impacts older people’s effort expectancy of technology, which in turn impacts older people’s intention to use videoconferencing. Since self-efficacy and effort expectancy can be quite comparable [45], we executed as a kind of sensitivity analysis the path analysis without effort expectancy, which showed a significant association between self-efficacy and intention to use technology.

Self-efficacy and digital literacy was also identified as the most prevalent theme during the observations in phase 2. Four additional themes were identified that could help us understand older people’s readiness to receive telehealth: “obstacles to using technology,” “prior experience and frequency in use,” “sources of support and facilitating conditions,” and “performance expectancy.”

Two of the themes, self-efficacy and performance expectancy, were also part of our theoretical framework and path analysis on intention to use technology. Additionally, the construct effort expectancy was observed within the theme “self-efficacy and digital literacy.” The qualitative results indicate that older people’s use of technology is associated with the themes we found. It is interesting to test in future research whether these themes (eg, facilitating conditions, prior experience, task structure) are also associated with older people’s intention to use. In our path analysis, frequency of internet use did not appear to be a significant predictor of intention to use, but perhaps (prior) experience with other types of technology does have a significant association with older people’s intention to use. Figure 3 summarizes the findings of both the constructs of the path analysis (phase 1) and the themes and subthemes derived from the observations (phase 2).

### Integration With Prior Research

Figure 3 illustrates how the themes found in phase 2 are related with the subthemes. On the basis of prior research, one can argue that there are more interactions within this framework to explore. Sponselee [46], for example, describes that family support positively impacts users’ frequency of use. Subsequently, the frequency of use might positively impact older people’s self-efficacy since performance accomplishments and successes that raise mastery expectations are seen as the strongest methods of increasing self-efficacy [47]. Another association that might be useful to explore in further research is that between facilitating conditions and obstacles. During observations, we learned that when participants had to overcome obstacles to technology use, they reached out to various sources of support, which differed from person to person depending on the level of the individuals’ facilitating conditions.

Additionally, older people’s motivation to start using or purchase technology can substantially differ, illustrated by one of our participant who explained her motivation to purchase a computer: “I had to do financial matters, and at that moment, I took a computer.” This finding is in line with Peek [48] who concludes that improving older people’s acceptance of technology requires, among other things, an understanding of the specific needs and circumstances of the targeted individual. Peek [48] also emphasizes that the acceptance of technology by older people is a dynamic process; specific events that occur in an individual’s life can trigger the need of using technology.

Regarding the predictors we found, effort expectancy and performance expectancy were already known from the TAM [11] and UTAUT [12], as well as observed in other health care-related studies [8,17] and from health care providers [49]. What this study adds to the TAM [11] and UTAUT [12] is that older people’s intention to use videoconferencing also can be predicted by their perceived privacy or security. Furthermore, the multilevel regression shows that effort expectancy was predicted by self-efficacy, and performance expectancy was predicted by effort expectancy. Our findings concur with those from other research studies [50] that emphasized the shortcomings of the common TAMs with regard to obtaining a deep understanding of older people’s readiness for using technology. By using a mixed-method design, this study shows (in phase 2) how some of the constructs of the path analysis regarding older people’s intention to use videoconferencing (ie, performance expectancy, effort expectancy, and self-efficacy) also play a role in the day-to-day situation of older people when they are using technology.

Contrary to the findings of prior research [51-53], “subjective health status” in our study was not found to be a relevant theme for older people’s technology use, neither in their intention to use videoconferencing, as shown in Figure 2, nor during the observations. Moreover, the performance of the path analysis model enhanced considerably (on the basis of the AIC) after we excluded subjective health status in Figure 3. Zimmer and Chappell [54] drew a comparable conclusion. In their study, older people’s self-assessed health was not significantly associated with their receptivity to new technology. Thus, caution is required when linking older people’s subjective health status to their intention to use technology.

Within the theme “sources of support and facilitating conditions,” the significant role of family members was identified. This observation is aligned with the prior research of Luijkx et al [55]. In this interview-study, Luijkx and colleagues emphasized the importance of including family members when implementing technology into the lives of older people and described that especially grandchildren can positively influence the acceptance of technology. Peek and colleagues [51] added that older people sometimes are afraid to burden their children with technology-related questions. This could also have played a part in one of our observations, in which an older person told us: “My children once said, ‘just put all those names here’ but I don’t have a clue of the meaning of all this.” When it comes to the role of family members, we observed an ambivalent mechanism; in accordance to prior research, family members can generate enthusiasm for using technology among older people, but at the same time, family members can also hamper the digital literacy of older people by taking over their technological tasks, which foregoes the opportunity for older people to become more skilled with using the technology.

### Study Limitations and Strengths

Our sampling strategy might have been a study limitation. Since the total number of potential respondents was not known, we could not measure a response rate and may have thus missed this indicator of representativeness. Only for those respondents who were recruited via the e-panel (n=186) we could, resulting in a response rate 9.30% (186/2000), which is low [56]. In the Netherlands, only 5% of the community-dwelling older people uses videoconferencing, according to a poll in 2016 [2]. Perhaps, the lack of experience of the remaining 95% of the population hampered their enthusiasm to participate in the survey about videoconferencing.

The online respondents of our study represent the largest part of our sample (72.3%). As a result, our sample was biased by a higher percentage of internet users compared with the general Dutch population of older people, in which 74% of the 65- to 75-year-old population and 34% of the population over 75 years of age occasionally used the internet in 2012 [57]. In our sample, about 94% of the 65- to 75-year-old population and 89% of the population over 75 years of age had experience with using the internet (at least) occasionally. Additionally, 46.5% of our sample completed higher education, which does not reflect the percentage of highly educated older people in Dutch society, namely 17% in 2012 [58]. We do not know whether the interactions we found in phase 1 would have also been found if the distribution of our sample was less skewed toward highly educated older people with a relatively high amount of technology experience. The sample skewness, however, only applies to phase 1. To observe both older people who possibly already had more digital skills or technology experience and those who did not, in phase 2, we carefully selected our participants, resulting in a sample in which approximately half of the participants did not use a computer (n=7) and the other half did use a computer (n=8).

We believe that our study strength lies in the triangulation of two methods, which helped us to gain a deep understanding of the often-used constructs in technology-acceptance models. Moreover, we noted the added value of the observation method (instead of interviews) to gain an understanding of technology use. With 9 of the 15 participants, a situation occurred in which they misjudged their digital skills; they overestimated or underestimated their skills, and as a result, they could or could not complete a technology task in contrast to their prior expectations. Our method of observations was not hindered by this form of recall bias, whereas it might have spoiled our results if we had chosen a different method, such as interviews.

### Implications for Practice or Education and Future Research

#### Education or Training

Older people’s intention to use technology is directly predicted by their effort expectancy, performance expectancy, and perceived privacy or security. Furthermore, self-efficacy and digital literacy appeared to play an important role in the day-to-day use of technology by older people and increase their effort expectancy. Therefore, we recommend addressing these concepts in technology training for older people to be given by nurses or other educators. We believe that in starting with increasing older people’s self-efficacy, their effort expectancy and intention to use will follow. In the literature, performance accomplishments, which are successes that raise mastery expectations, are seen as the strongest method of increasing self-efficacy [47]. As mentioned, during our observations, several participants discovered their ability to accomplish a technological task contrary to their prior expectations. In training, similar practices could be organized with the aim of giving older people the opportunity to achieve performance accomplishments. This practice will be the strongest intervention to raise their self-efficacy and as a result their intention and capacity to use technology.

The second strongest source of self-efficacy is vicarious experience, namely seeing others accomplish difficult situations [47]. During training, older people’s self-efficacy will most likely increase as technological tasks are repeatedly shown to be achievable by a variety of models. Although this modeling strategy is less effective than personal accomplishment, it may be suited for training purposes by letting participants observe each other executing technological tasks.

A final thought for supporting older people in technology use comes from our observation that some of our participants kept very strictly to the skills that they had learned and became nervous about trying anything outside of their skill set. One can argue about the most appropriate way of learning: (1) providing very specific concrete instructions focused on specific applications or devices or (2) starting from more general technological competencies that could perhaps be applied to a variety of situations, applications, or devices. Hickman et al [59] show that, if the goal is to support learning, “guided attention training” works better for older people than “guided action training,” in which participants are told exactly what to do at every step. More research, similar to Hickman et al [59], is needed to learn more about what approach may work best.

Above, we take the perspective that barriers to technology use are a result of a lack of self-efficacy among the end users, in this study of older people. However, the lack of self-efficacy can also be the result of an inappropriate design of the technology. Tsai and colleagues [60] showed that when a new technology is easy to use, a lack of self-efficacy was not a strong barrier for older people to use this technology. So, besides developing adequate training programs for older people, it is useful to think of designing appropriate technology that is easy to use.

#### Future Research

To test the suggestions above, more research with regard to older people’s technology use is required. Our overarching aim was to place older people in a better position to benefit from new ways of health care provision. In this study, we gained a deep understanding of older people’s day-to-day use of technology, which can be used as a basis for training development. Research into older people’s beliefs regarding their capacities in using health care technology using a pretest-posttest setup, before and after a training, might be a logical next step in research.

### Conclusions

This study shows that older people’s intention to use videoconferencing is directly predicted by their performance expectancy, effort expectancy, and perceived privacy or security. Additionally, self-efficacy significantly impacts older people’s effort expectancy, which subsequently impacts older people’s performance expectancy of videoconferencing. In the day-to-day situation, older people experience all kinds of obstacles when using digital technology. Self-efficacy and digital literacy appeared to be the most important theme that plays a role in their technology use and overcoming barriers. Overcoming barriers to technology use is necessary to be able to make use of the new ways of receiving health care involving digital technology.

## Appendix

Multimedia Appendix 1 Literature review, search terms, and hits.

## Abbreviations

AIC: Akaike Information Criterion
OR: odds ratio
TAM: Technology Acceptance Model
UTAUT: Unified Theory of Acceptance and Use of Technology

## References

[1] Schippers EI, van Rijn M (2014). Rijksoverheid.

[2] Krijgsman J, Peeters J, Waverijn G, Lettow B, Hoek L (2016). Nivel.

[3] Olson KE, O'Brien MA, Rogers WA, Charness N (2011). Diffusion of technology: frequency of use for younger and older adults. Ageing Int.

[4] Sackmann R, Winkler O (2013). Technology generations revisited: the internet generation. Gerontechnology.

[5] Holden RJ, Karsh B (2010). The technology acceptance model: its past and its future in health care. J Biomed Inform.

[6] van Houwelingen CT, Moerman AH, Ettema RG, Kort HS, Ten Cate O (2016). Competencies required for nursing telehealth activities: a Delphi-study. Nurse Educ Today.

[7] Heerink M, Kröse B, Wielinga BV, Evers V (2008). Enjoyment intention to use and actual use of a conversational robot by elderly people.

[8] de Veer AJ, Peeters JM, Brabers AE, Schellevis FG, Rademakers JJ, Francke AL (2015). Determinants of the intention to use e-Health by community dwelling older people. BMC Health Serv Res.

[9] Pan S, Jordan-Marsh M (2010). Internet use intention and adoption among Chinese older adults: From the expanded technology acceptance model perspective. Comput Hum Behav.

[10] Nayak LU, Priest L, White AP (2010). An application of the technology acceptance model to the level of Internet usage by older adults. Univ Access Inf Soc.

[11] Venkatesh V, Davis FD (2000). A theoretical extension of the technology acceptance model: four longitudinal field studies. Manage Sci.

[12] Venkatesh V, Thong JY, Xu X (2012). Consumer acceptance and use of information technology: Extending the unified theory of acceptance and use of technology. MIS Q.

[13] Bhattacherjee A, Sanford C (2009). The intention–behaviour gap in technology usage: the moderating role of attitude strength. Behav Inf Technol.

[14] Tsai T, Chang H, Chen Y, Chang Y (2017). Determinants of user acceptance of a specific social platform for older adults: an empirical examination of user interface characteristics and behavioral intention. PLoS One.

[15] Hoque R, Sorwar G (2017). Understanding factors influencing the adoption of mHealth by the elderly: an extension of the UTAUT model. Int J Med Inform.

[16] Cajita MI, Hodgson NA, Budhathoki C, Han H (2017). Intention to use mHealth in older adults with heart failure. J Cardiovasc Nurs.

[17] Tsai C (2014). Integrating social capital theory, social cognitive theory, and the technology acceptance model to explore a behavioral model of telehealth systems. Int J Environ Res Public Health.

[18] Tsai T, Wong AM, Hsu C, Tseng KC (2013). Research on a community-based platform for promoting health and physical fitness in the elderly community. PLoS One.

[19] Wang L, Rau PP, Salvendy G (2011). Older adults' acceptance of information technology. Educ Gerontol.

[20] Braun MT (2013). Obstacles to social networking website use among older adults. Comput Hum Behav.

[21] Wang Q, Sun X (2016). Investigating gameplay intention of the elderly using an Extended Technology Acceptance Model (ETAM). Technol Forecast Soc.

[22] Sintonen S, Immonen M (2013). Telecare services for aging people: assessment of critical factors influencing the adoption intention. Comput Hum Behav.

[23] Shah SG, Barnett J, Kuljis J, Hone K, Kaczmarski R (2013). Factors determining patients' intentions to use point-of-care testing medical devices for self-monitoring: the case of international normalized ratio self-testing. Patient Prefer Adherence.

[24] Greene JC, Caracelli VJ, Graham WF (1989). Toward a conceptual framework for mixed-method evaluation designs. Educ Eval Policy An.

[25] Harrell FE, Lee KL, Mark DB (1996). Multivariable prognostic models: issues in developing models, evaluating assumptions and adequacy, and measuring and reducing errors. Stat Med.

[26] Czaja SJ, Charness N, Dijkstra K, Fisk AD, Rogers WA, Sharit J (2006). Create Center.

[27] Czaja SJ, Charness N, Dijkstra K, Fisk AD, Rogers WA, Sharit J (2006). Create Center.

[28] Chang IC, Hsu HM (2012). Predicting medical staff intention to use an online reporting system with modified unified theory of acceptance and use of technology. Telemed J E Health.

[29] Gagnon MP, Orruño E, Asua J, Abdeljelil AB, Emparanza J (2012). Using a modified technology acceptance model to evaluate healthcare professionals' adoption of a new telemonitoring system. Telemed J E Health.

[30] Janssen KJ, Donders AR, Harrell FE, Vergouwe Y, Chen Q, Grobbee DE, Moons KG (2010). Missing covariate data in medical research: to impute is better than to ignore. J Clin Epidemiol.

[31] Rubin DB (1996). Multiple imputation after 18+ years. J Am Stat Assoc.

[32] Terwee CB, Bot SD, de Boer MR, van der Windt DA, Knol DL, Dekker J, Bouter LM, de Vet HC (2007). Quality criteria were proposed for measurement properties of health status questionnaires. J Clin Epidemiol.

[33] Duncan OD (1966). Path analysis: sociological examples. Am J Sociol.

[34] Akaike H (1974). A new look at the statistical model identification. IEEE Trans Automat Contr.

[35] Hsieh HF, Shannon SE (2005). Three approaches to qualitative content analysis. Qual Health Res.

[36] Bunniss S, Kelly DR (2010). Research paradigms in medical education research. Med Educ.

[37] Bowen GA (2016). Grounded theory and sensitizing concepts. Int J Qual Meth.

[38] McFarland DJ, Hamilton D (2006). Adding contextual specificity to the technology acceptance model. Comput Hum Behav.

[39] Central Committee on Research Involving Human Subjects http://www.ccmo.nl/en/your-research-does-it-fall-under-the-wmo.

[40] Bandura AE, Ramachaudran VS (1994). Self-efficacy. Encyclopedia of Human Behavior, Volume 4.

[41] Eshet-Alkalai Y (2004). Digital literacy: a conceptual framework for survival skills in the digital era. J Educ Multimed Hypermedia.

[42] Gitlow L (2014). Technology use by older adults and barriers to using technology. Phys Occup Ther Geriatr.

[43] Gatto SL, Tak SH (2008). Computer, internet, and e-mail use among older adults: benefits and barriers. Educ Gerontol.

[44] Goodhue DL, Thompson RL (1995). Task-technology fit and individual performance. MIS Q.

[45] Venkatesh V, Davis FD (1996). A model of the antecedents of perceived ease of use: development and test. Decision Sci.

[46] Sponselee A (2013). Acceptance and Effectiveness of Telecare Services From the End-User Perspective [Doctoral Thesis].

[47] Bandura A (1977). Self-efficacy: toward a unifying theory of behavioral change. Psychol Rev.

[48] Peek S (2017). Understanding Technology Acceptance by Older Adults Who Are Aging in Place [Doctoral Thesis].

[49] van Houwelingen CT, Barakat A, Best R, Boot WR, Charness N, Kort HS (2015). Dutch nurses' willingness to use home telehealth: implications for practice and education. J Gerontol Nurs.

[50] Berge M (2016). Telecare acceptance as sticky entrapment: a realist review. Gerontechnology.

[51] Peek ST, Wouters EJ, van Hoof J, Luijkx KG, Boeije HR, Vrijhoef HJ (2014). Factors influencing acceptance of technology for aging in place: a systematic review. Int J Med Inform.

[52] Chen K, Chan AH (2014). Gerontechnology acceptance by elderly Hong Kong Chinese: a senior technology acceptance model (STAM). Ergonomics.

[53] Bürmann Genannt Siggemann C, Mensing M, Classen T, Hornberg C, Terschüren C (2013). Specific health status has an impact on the willingness to use telemonitoring: data from a 2009 health survey in North Rhine-Westphalia, Germany. Telemed J E Health.

[54] Zimmer Z, Chappell NL (1999). Receptivity to new technology among older adults. Disabil Rehabil.

[55] Luijkx K, Peek S, Wouters E (2015). “Grandma, you should do it-it's cool” older adults and the role of family members in their acceptance of technology. Int J Environ Res Public Health.

[56] Fincham JE (2008). Response rates and responsiveness for surveys, standards, and the Journal. Am J Pharm Educ.

[57] Centraal Bureau voor de Statistiek https://www.cbs.nl/en-gb/news/2013/21/one-third-of-over-75s-use-the-internet.

[58] Centraal Bureau voor de Statistiek Bevolking; hoogst behaald onderwijsniveau; geslacht, leeftijd en herkomst Society; highest completed education; gender, age and origin.

[59] Hickman JM, Rogers WA, Fisk AD (2007). Training older adults to use new technology. J Gerontol B Psychol Sci Soc Sci.

[60] Tsai HS, Shillair R, Cotten SR, Winstead V, Yost E (2015). Getting grandma online: are tablets the answer for increasing digital inclusion for older adults in the US?. Educ Gerontol.

