# Supplementary material for: Understanding Older People’s Readiness for Receiving Telehealth: Mixed-Method Study
Source: J Med Internet Res. 2018 Apr 6;20(4):e123. doi: 10.2196/jmir.8407 (PMC5910535; doi:10.2196/jmir.8407)
Supplement: Multimedia Appendix 1 [file jmir_v20i4e123_app1.pdf]

Appendix 1. Literature review, search terms and hits

Source	Search terms	Hits	Selected for use
**PubMed**	((older adults[title/abstract] OR older people[title/abstract] OR seniors[title/abstract] OR elderly[title/abstract]) AND (technology[title/abstract])) AND intention[title/abstract]) AND (factors [title/abstract] OR determinants[title/abstract] OR associations[title/abstract])))	22	6 [1]
**CINAHL**	(AB older adults OR AB older people OR AB seniors OR AB elderly) AND (AB technology) AND (intention) AND (AB factors OR AB determinants OR AB associations)	8	1 [2]
**ScienceDirect**	TITLE-ABSTR-KEY("older adults") or TITLE-ABSTR-KEY(elderly) or TITLE-ABSTR-KEY("older people") or TITLE-ABSTR-KEY(seniors) and TITLE-ABSTR-KEY(technology) and TITLE-ABSTR-KEY(intention) and TITLE-ABSTR-KEY(factors) or TITLE-ABSTR-KEY(determinants) or TITLE-ABSTR-KEY(associations)	23	2 [3]
**Google Scholar**	allintitle: (older OR elderly OR seniors) AND technology AND (intention) AND (factors OR determinants OR associations)	0	0
**Web of Science**	TS=(older adults OR seniors OR elderly OR older people) AND TS=(technology) AND TS=(intention) AND TS=(factors OR determinants OR associations)	84	10 [4]
**Scopus**	(TITLE-ABS-KEY (older AND people) OR TITLE-ABS-KEY (older AND adults) OR TITLE-ABS-KEY (elderly) OR TITLE-ABS-KEY (seniors)) AND (TITLE-ABS-KEY (technology)) AND TITLE-ABS-KEY (intention) AND (TITLE-ABS-KEY (factors) OR TITLE-ABS-KEY (determinants) OR TITLE-ABS-KEY (associations)) AND PUBYEAR > 2006 AND (LIMIT-TO (LANGUAGE , "English")) AND (LIMIT-TO (DOCTYPE , "ar"))	95	7 [5]
**PsycINFO**	((Older adults or Older people or Elderly or Seniors) and Technology and (Intention) and (Factors or Determinants or Associations)).tw.	17	2 [6]
		Selected	29
		Duplicates	18
		Finnaly included	11

1. Tsai et al. (2017) [14]; Hoque & Sorwar (2017) [15]; Cajita et al. (2017) [16]; de Veer et al. (2015) [8]; Tsai (2014) [17]; Tsai et al. (2013) [18]
2. Hoque & Sorwar (2017) [15]; Wang et al. (2011) [19]
3. Hoque & Sorwar (2017) [15]; Braun (2013) [20]
4. Tsai et al. (2017) [14]; Hoque & Sorwar (2017) [15]; Wang & Sun (2016) [21]; de Veer et al. (2015) [8]; Tsai (2014) [17]; Sintonen & Immonen (2013) [22]; Braun (2013) [20]; Tsai et al. (2013) [18]; Shah et al. (2012) [23]; Wang et al. (2011) [19]
5. Tsai et al. (2017) [14]; Hoque & Sorwar (2017) [15]; Wang & Sun (2016) [21]; de Veer et al. (2015) [8]; Tsai (2014) [17]; Braun (2013) [20]; Wang et al. (2011) [19]
6. Braun (2013) [20]; Wang et al. (2011) [19]
